# Sensory Coding and Sensitivity to Local Estrogens Shift during Critical Period Milestones in the Auditory Cortex of Male Songbirds

**DOI:** 10.1523/ENEURO.0317-17.2017

**Published:** 2017-12-12

**Authors:** Daniel M. Vahaba, Matheus Macedo-Lima, Luke Remage-Healey

**Affiliations:** 1 Neuroscience and Behavior Graduate Program; 2 Center for Neuroendocrine Studies, University of Massachusetts, Amherst, MA 01003; 3 Department of Psychological & Brain Sciences, University of Massachusetts, Amherst, MA 01003; 4 CAPES Foundation, Ministry of Education of Brazil, 70040-020, Brazil DF

**Keywords:** Auditory cortex, communication processing, critical periods, lateralization, neuroestrogens, songbird

## Abstract

Vocal learning occurs during an experience-dependent, age-limited critical period early in development. In songbirds, vocal learning begins when presinging birds acquire an auditory memory of their tutor’s song (sensory phase) followed by the onset of vocal production and refinement (sensorimotor phase). Hearing is necessary throughout the vocal learning critical period. One key brain area for songbird auditory processing is the caudomedial nidopallium (NCM), a telencephalic region analogous to mammalian auditory cortex. Despite NCM’s established role in auditory processing, it is unclear how the response properties of NCM neurons may shift across development. Moreover, communication processing in NCM is rapidly enhanced by local 17β-estradiol (E2) administration in adult songbirds; however, the function of dynamically fluctuating E_2_ in NCM during development is unknown. We collected bilateral extracellular recordings in NCM coupled with reverse microdialysis delivery in juvenile male zebra finches (*Taeniopygia guttata*) across the vocal learning critical period. We found that auditory-evoked activity and coding accuracy were substantially higher in the NCM of sensory-aged animals compared to sensorimotor-aged animals. Further, we observed both age-dependent and lateralized effects of local E_2_ administration on sensory processing. In sensory-aged subjects, E_2_ decreased auditory responsiveness across both hemispheres; however, a similar trend was observed in age-matched control subjects. In sensorimotor-aged subjects, E_2_ dampened auditory responsiveness in left NCM but enhanced auditory responsiveness in right NCM. Our results reveal an age-dependent physiological shift in auditory processing and lateralized E_2_ sensitivity that each precisely track a key neural “switch point” from purely sensory (pre-singing) to sensorimotor (singing) in developing songbirds.

## Visual Abstract

**Figure:**
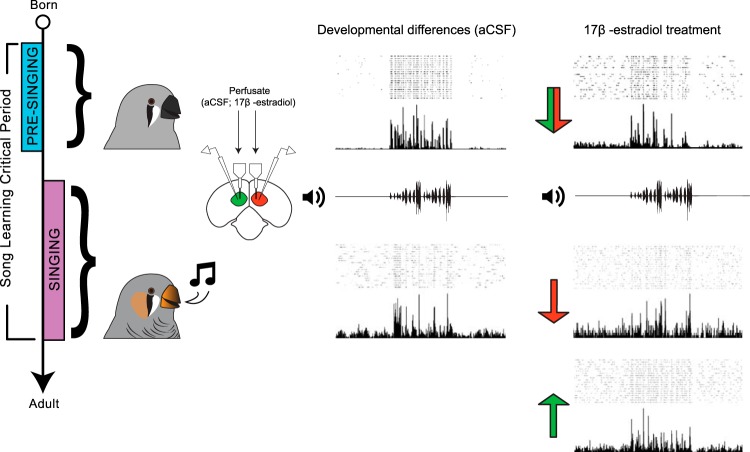

## Significance Statement

Vocal communication, such as language and birdsong, is learned during an age-limited critical period early in development. Initially, infants and songbirds exclusively listen to memorize their native tongue before producing nascent vocalizations. We show that the transition from pre-singing to vocalizing in developing songbirds is accompanied by a large shift in auditory gain and coding in cortical neurons. Further, whereas estrogens generally improve hearing in adulthood, we found that brain estrogens either enhanced or diminished auditory responsiveness depending on both critical period phase and cerebral hemisphere. Our findings therefore highlight a neural transition in auditory processing and lateralized hormone sensitivity at a key stage in development, and similar mechanisms could be relevant for speech processing and language acquisition in humans.

## Introduction

Critical periods are windows of heightened experience-dependent neuroplasticity in which early sensory input shapes neural circuits and behaviors. Critical period research has historically focused on how sensory exposure or deprivation drive cortical and behavioral shifts in development (Lorenz, 1937; Wiesel and Hubel, 1963; Bolhuis, 1991; Hensch, 2005). Some critical periods for learned behaviors, such as vocal communication, shift from being purely sensory (auditory) to an active sensorimotor phase (vocal production, exploration, and refinement; Kuhl, 2010). Such behavioral transitions are likely accompanied by neural changes in sensory processing. Relatively little is known about factors that change during vocal communication learning, however, as experience-dependent learned vocal communication (vocal learning) is found in only a handful of animal species, including humans and songbirds (Petkov and Jarvis, 2012).

**Figure 1.**
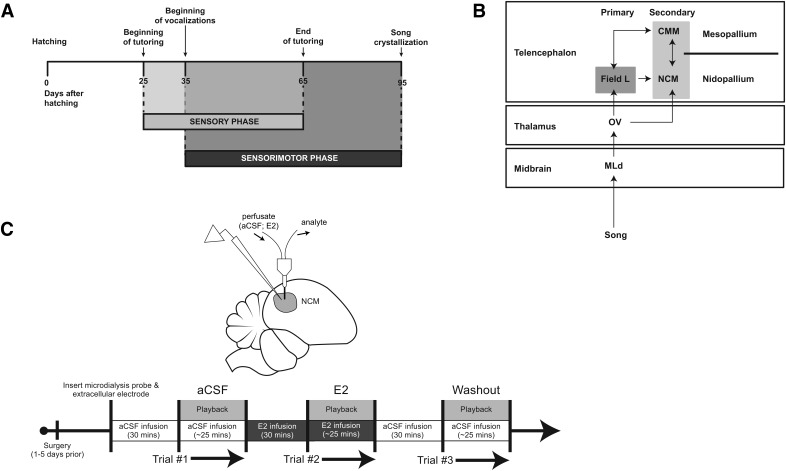
**Critical period timeline, avian auditory circuit, and experimental paradigm. *A***, The critical period for song learning unfolds across a 3 month timespan. Whereas some songbird species begin song learning and recognition at embryonic stages of development (Colombelli-Négrel et al., 2012), zebra finch sensory learning begins at 25 dph (Clayton, 2013). Autogenous song production can occur as early as 35 dph (typically closer to 40 dph; personal observation), and initially overlaps with the sensory learning phase, until 65 dph when sensorimotor-only learning continues as birds begin to refine their developing subsong until eventual song crystallization (∼100 dph). Timeline adapted after Clayton (2013). ***B***, Schematic of the avian ascending auditory neural circuit. After sounds are first processed in upstream peripheral and brainstem auditory regions, communication is encoded within the midbrain nucleus MLd (dorsal part of the lateral mesencephalic nucleus), which sends projections to the thalamic nucleus ovoidalis (Ov). Ov sends projections primarily to Field L, comparable to mammalian primary auditory cortex, as well as to NCM (Vates et al., 1996). Secondary auditory cortex regions NCM (caudomedial nidopallium) and CMM (caudomedial mesopallium) are reciprocally connected and receive afferent projections from Field L. ***C***, Experimental setup and paradigm. Top: *In vivo* microdialysis and extracellular electrophysiology schematic. A microdialysis cannula was first descended into NCM (∼1.10 mm ventral; light gray circular region). Afterward, a carbon-fiber electrode was placed within the proximate region of perfusate diffusion. Bottom: Experimental timeline. aCSF, artificial cerebrospinal fluid; E_2_, 17β-estradiol.

In some songbird species, such as zebra finches (*Taeniopygia guttata*), males are the exclusive vocal learners (Immelmann, 1969). Males learn song during two developmental phases (Fig. 1*A*). In the sensory phase, birds acquire an auditory memory of their tutor’s song, and then slowly refine their burgeoning vocalizations to approximate this tutor memory during the sensorimotor phase (Mooney, 2009). Research on the neural circuitry of vocal learning has largely explored song production premotor and cortico-basal ganglia circuits (Roberts et al., 2012; Brainard and Doupe, 2013). While auditory processing is necessary for song learning (Thorpe, 1954; Konishi, 1965), far less is known about the contribution of the auditory cortex during song learning in early development.

The caudomedial nidopallium (NCM; Fig. 1*B*) is key for auditory processing. NCM receives projections from primary cortical thalmo-recipient Field L, and is considered the avian analog of the mammalian secondary auditory cortex (Vates et al., 1996; Wang et al., 2010). NCM is important for both processing species-specific vocal communication (Mello et al., 1992; Theunissen et al., 2004) and auditory memory consolidation (Chew et al., 1995; London and Clayton, 2008; but see Canopoli et al., 2014). Further, much like the neural circuits for human language processing, NCM’s role in auditory memory encoding and processing appears to be lateralized (Avey et al., 2005; Moorman et al., 2012, 2015; De Groof et al., 2013). Despite this clear role in auditory function, it is unclear how NCM’s response properties shift across the vocal learning critical period.

In zebra finches, auditory behavioral perception and discrimination are adult-like as early as ∼30 days posthatching (dph; Braaten et al., 2006). Studies on developmental changes in NCM neurophysiology have focused on the putative opening and closing of the sensory phase (20 and ∼30–35 dph, respectively; Böhner, 1990), but not beyond (Stripling et al., 2001; Miller-Sims and Bottjer, 2014). While there are subtle differences between juvenile age groups for song selectivity, auditory preferences and response magnitude at 35 dph are comparable to adults. Similarly, Jin and Clayton (1997) found that NCM neuronal cell density is also similar to adults at 20 and 30 dph. To date, changes in communication processing in auditory forebrain outside of the sensory phase has been limited to immediate-early gene studies on 45-dph zebra finches (Bailey and Wade, 2003, 2005) and physiology studies on tutor song selectivity at ∼22 or ∼60 dph (Adret et al., 2012; Yanagihara and Yazaki-Sugiyama, 2016, respectively).

Circulating estrogens fluctuate across the critical period in several songbird species (Pröve, 1983; Weichel et al., 1986; Marler et al., 1988; but see Adkins-Regan et al., 1990) and predict vocal learning success (Marler et al., 1987), as in humans (Wermke et al., 2014). Estradiol levels in NCM gradually increase over the critical period, and also acutely in response to single tutoring bouts in juvenile male zebra finches (Chao et al., 2015). In adult songbirds, both circulating (references) and brain-derived estrogens (neuroestrogens; namely 17β-estradiol [E_2_]; Remage-Healey et al., 2010; Remage-Healey and Joshi, 2012) generally enhance complex communication encoding within telencephalic auditory brain regions, including NCM. Unlike other avian auditory forebrain nuclei that are devoid of estrogen synthase (Field L and CMM; Fig. 1*B*), NCM is highly enriched with aromatase (Saldanha et al., 2000; Peterson et al., 2005). Moreover, while ascending auditory circuits are conserved across Aves, aromatase is uniquely found within the NCM of vocal learners (Metzdorf et al., 1999; Silverin et al., 2000). Together, these observations suggest that fluctuating neuroestrogens in NCM may dynamically influence auditory processing in development.

We tested two hypotheses, that (1) auditory responsiveness to natural communication signals in NCM changes across the critical period for vocal learning; and (2) NCM auditory responsiveness and coding are rapidly modulated by changes in local estrogens.

## Materials and Methods

### Subjects

All animal procedures were performed in accordance with the Institutional Animal Care and Use Committee at the University of Massachusetts Amherst. Male zebra finches (*N* = 31 birds; *n* = 26 for estradiol experiments; *n* = 5 for control recordings) were obtained from our breeding colonies, ranging in age from 25 to 95 dph. Hemisphere was considered the unit of replication, as NCM is a bilateral structure with no direct reciprocal connections between hemispheres (Vates et al., 1996). Subjects were initially binned by age reflecting the different critical period phases for song learning (Fig. 1*A*): sensory, 25–34 dph (left = 4; right = 5); sensory/sensorimotor: 40–64 dph (left = 13; right = 8); and sensorimotor: 65–95 dph (left = 5; right = 3). Zebra finches begin displaying overt sexually dimorphic plumage at ∼40 dph. For subjects <40 dph or that did not have male features (black striations, brown badge feathers, orange cheeks, etc.), DNA was extracted from whole blood, and PCR was run to determine sex (see below). Subjects were raised in mixed-sex breeding colonies in a 14:10 light:dark cycle. Once selected for the experiment, subjects were housed in an acoustic isolation chamber with a nonrelated adult companion female. For presinging 25- to 34-dph subjects, either the experiment was conducted the same day as the surgery, or subjects were isolated with a companion female for 1 d before the experiment. For 40- to 95-dph birds, subjects were cohoused with a companion female for 2–7 days before the experiment to capture birds’ own song (BOS), which was recorded using Sound Analysis Pro (Tchernichovski et al., 2000) via an omnidirectional microphone (Countryman) inside a sound-attenuation chamber (Eckel Acoustics).

### Sex determination PCR

For juvenile birds without discernable male features (<35 dph), whole blood was obtained from the ulnar vein, and DNA was subsequently extracted using a commercially available kit (QIAmp DNA Mini Kit; Qiagen #51304). Purified DNA was subsequently used for PCR using a set of degenerate primers linked to the Z- and W-chromosomes (Griffiths et al., 1998). Amplified PCR product was then visualized alongside a negative control (water) and both adult male and female positive controls on a 2% agarose gel using electrophoresis. Subjects with two bands separated by 36 bp were excluded from the study (indicating presence of W chromosome; thus females), and subjects showing a single band (indicating no W chromosome) were retained for the experiment.

### Surgery

Surgery was performed 1–5 d before the experiment for most subjects (Fig. 1*C*; surgery was conducted the day of recordings in 2 birds). Animals were food deprived for 30 min before an intramuscular injection of Equithesin (30–40 µL), and 20 min later, birds were wrapped in a cloth jacket and secured to a custom designed surgical stereotaxic apparatus (45° head angle; Herb Adams Engineering) with a heating pad underneath (36°C). Scalp feathers were removed, and a 20 µL subcutaneous injection of lidocaine (2% in ethanol; Sigma-Aldrich) was administered under the scalp. The scalp was then resected, and a positioning-needle was placed just posterior to the midsagittal sinus bifurcation (MSB) and used as a 0-point anatomic reference. The skull was then marked at the anterior-most extent of NCM: rostral = –1.20 mm and lateral/medial = 0.90 mm, relative to the MSB. This marking provided a site for microdialysis probe implantation on the day of recording (see below) alongside recording electrodes immediately adjacent (caudal) into NCM. A silver wire was implanted between skull leaflets over the cerebellum to serve as a reference ground. A head-post was then affixed to the bird using cyanoacrylate and dental cement. After surgery, birds were placed in a recovery cage on a heating pad (36°C) with available food and water until they awoke from the anesthetic. After recovery, birds were given an oral administration of Meloxicam (1 µL/g weight; 0.1 mg/mL) and returned to their acoustic isolation chamber in a separate cage from the companion female.

### Anesthetized extracellular electrophysiology and acute estradiol treatment

On the day of the experiment, subjects were food deprived for 30 min before initial anesthetic injections. After 30 min of food deprivation, 90–100 µL of 20% urethane was evenly administered across three injections separated by 45 min each. Once the subject was anesthetized, subjects were brought to the recording room and affixed to a custom head-post stereotaxic apparatus (45° head angle; Herb Adam Engineering). A small fenestra was made over one hemisphere of NCM and the dura was resected. A microdialysis probe (CMA-7; Harvard Apparatus) was first inserted just anterior to the intersecting point of NCM (as marked by the prior surgery; ∼1.10 mm ventral; Fig. 1*C*), and artificial cerebrospinal fluid (aCSF) was perfused at 2 μl/min using a syringe pump (PHD 2000; Harvard Apparatus). Implanting microdialysis probes creates an acute injury in the brain, which induces a local increase in glial aromatase after 24 h in male zebra finches (Saldanha et al., 2013). Here, microdialysis probes were implanted for no longer than 4 h, so it is unlikely that injury-induced glial aromatase influenced NCM properties within the time course of the current experiments.

After the probe was inserted, a carbon fiber electrode (CarboStar-1; Kation) was placed within the proximity of the microdialysis probe, and a recording site was found using search stimuli (Fig. 1*C*). A recording site was determined as being within NCM based on its: (1) anatomic coordinates (0.80–1.40 mm ventral) and (2) spontaneous and stimulus-evoked activity using a set of nonexperimental stimuli (search stimuli; see below).

After at least 30 min of aCSF infusion had elapsed, the first of three trials began (Fig. 1*C*). Each trial included 20 repeats of each stimulus with an interstimulus interval of 10 ± 2 s (experimental stimuli; see below), lasting ∼25 min. After the end of the first playback trial, 17β-estradiol (E_2_; 30 μg/mL [110 μM]; dose based on similar studies; Remage-Healey et al., 2010, 2012; Remage-Healey and Joshi, 2012; Pawlisch and Remage-Healey, 2015) was retrodialyzed for 30 min, and afterward, a new playback period (using the same stimuli as in trial 1) was presented while E_2_ was continuously infused. The same steps for E_2_ were repeated with aCSF alone for trial 3 as a washout period. At the end of the recording session, electrolytic lesions were performed at the recording site for later anatomic confirmation. The infusion/playback regimen in trials 1–3 was repeated when possible in the contralateral NCM (*n* = 12 of 26 subjects).

At the end of the experiment, birds were killed via rapid decapitation. Brains were removed and placed in a 20% sucrose-formalin solution at 4°C to allow for tissue fixation. Once fixed, brains were frozen in an embedding medium (O.C.T. compound; Tissue-Plus; Fisher HealthCare) and stored at –80°C until being sectioned at 45 μm and Nissl-stained for histologic verification of probe and electrode placement.

### Auditory stimuli and playback

Five unique conspecific songs and one white noise (WN) stimulus were used to initially identify auditory responsive recording sites typical of NCM (search stimuli). For playback trials, a unique set of experimental stimuli were used and included two novel conspecific male songs (CON1 and CON2; different from search stimuli CON), heterospecific song (Bengalese finch; HET), and WN. Bird’s own song (BOS) and temporally reversed BOS (REV-BOS) was used when available for 40- to 95-dph animals. If BOS was unavailable for a 40- to 95-dph subject (*n* = 4), an age-matched juvenile male conspecific song (JUV CON) and temporally reversed JUV CON (REV-JUV CON) was used instead. For all sensory-aged subjects, a 40-dph JUV CON and REV-JUV CON was presented in place of BOS and REV-BOS. All stimuli were ∼2 s in duration (two motif renditions of directed song with introductory notes; ∼1.7- to 2.4-s total duration), normalized to ∼70 dB (A-weighted) and bandpass filtered at 0.3–15 kHz using Adobe Audition. Each playback trial randomly presented 20 repetitions of each stimulus (15 repetitions initially for the first 3 subjects) with a randomly determined interstimulus interval of 10 ± 2 s between each stimulus. The average playback trial duration was ∼25 min.

### Data analysis

Multiunit electrophysiological recordings were analyzed offline using Spike2 (v.7.04, Cambridge Electronic Design). For each unique subject’s multiunit analysis, a voltage threshold to distinguish signal from noise was initially set based on Trial 1 and was maintained across all subsequent trials. Thresholds were set at least 2-fold above the noise-band of a given recording. Recordings were then analyzed by suprathreshold activity aligned to the playback of auditory stimuli. Stimulus-evoked firing frequency was defined as the total number of spikes (threshold crossings) 2 s after auditory stimulus onset divided by the number of trials (stimulus repeats), whereas spontaneous firing frequency was defined as the number of threshold crossings 2-s period before the onset of an auditory stimulus divided by the total number of trials. To account for firing variability across subjects, auditory responses were normalized using *z*-score transformations using the following equation:

$$z\text{-score}=\frac{\overset{¯}{S}-\overset{¯}{B}}{\sqrt{Var\left(S\right)+Var\left(B\right)-2Covar\left(S,B\right)}},$$

where *S* is the number of spikes during stimulus response (2 s, beginning at stimulus onset), and *B* is the number of spikes during baseline (2 s before stimulus onset). $\bar{S}$ and $\bar{B}$ represent the means of these values across all stimulus presentations for a given playback trial.

### Single-unit spike sorting

Although multiunit physiologic recordings provide information about population responses, we also isolated single neurons to investigate auditory responsiveness for cells with high signal-to-noise ratios. Isolating single units provides an increased sample size, reducing animal usage numbers and allowing us to track the response properties of single neurons (1–2 units per recording site) over time in response to estrogen modulation. To identify putative single neurons for analysis, Trial 1 multiunit recordings were sorted for large-amplitude single-unit templates based on wave form using default settings in Spike2 (*n* = 53 single units). Sorted single units were retained for analysis if they were distinctly clustered from noise or other units in a principal components analysis space and had an interspike interval (ISI) >1 ms (i.e., zero ISIs were within the 1-ms bin for all units; Fig. 3*A*). After sorting, each single unit was confirmed to be auditory responsive using visual inspection of peristimulus time histograms, as well as by paired *t* tests comparing each unit’s spontaneous and stimulus-evoked firing rates. Units that were statistically responsive (*p* < 0.05) to at least one auditory stimulus during Trial 1 were included. On average, each multiunit recording site yielded 1–2 distinct and auditory-responsive single units. Peak-to-trough wave form durations were measured to initially distinguish broad- versus narrow-spiking neurons (as in Schneider and Woolley, 2013; Yanagihara and Yazaki-Sugiyama, 2016); however, we did not observe cell type–specific descriptive effects. Also because of inferential statistical power limitations, we opted to group all single units in our analyses and disregard wave form classifications.

### Pattern classifier

A custom pattern classifier was developed in Python to assess reliability and discriminability of neuronal responses to different stimuli (similar to Caras et al., 2015; as in Lee et al., 2017). For each single-unit recording, the stimulus-evoked firing responses to the 6 different stimuli were compared iteratively. At the start of each run of the classifier, one trial of each stimulus was pseudorandomly selected as the template (6 templates). All remaining 19 trials for each stimulus (114 trials total) were compared one at a time to the templates using a similarity measure. This procedure was repeated 1000 times to generate a confusion matrix, which represents data in terms of actual versus predicted stimulus classification (Fig. 3*F*).

Before comparison, each response to a stimulus iteration was Gaussian filtered. The standard deviation (σ) of the filter was employed as a variable for each cell, i.e., the classifier was run with varying σ values of 1, 2, 4, 8, 16, 32, 64, 128, and 256 ms (1000 simulations for each). The filter that yielded the highest accuracy score was used for that cell. Templates and trials were correlated by using the R_corr_ method (Schreiber et al., 2003; Caras et al., 2015):

$$R_{corr}=\frac{{\vec{s}}_{trial}\;\cdot \;{\vec{s}}_{template}}{\left|{\vec{s}}_{trial}\right|\cdot \left|{\vec{s}}_{template}\right|},$$

where $\overset{\to }{s}$ represents the vectors of the trial and the template responses after filtering, which are dot-multiplied then divided by the product of their lengths. This calculation returns a value between 0 and 1, which represent total dissimilarity or total similarity, respectively. The stimulus type of the template that provided the highest R_corr_(trial, template) value was considered the predicted stimulus for the trial in analysis. Therefore, percentage accuracy scores were generated by how well each neuron’s firing pattern was predictive of the auditory stimulus.

The classifier output for each neuron was assessed statistically via a trial shuffling approach (Caras et al., 2015). Trials were stripped of stimulus labels, pseudorandomly shuffled and relabeled, essentially generating random responses to the stimuli. The pattern classifier was then run with this shuffled dataset. The distribution of the accuracies (means of diagonals in the confusion matrices) generated in each run of the original dataset was compared with the shuffled dataset via Cohen’s *d*. Cohen’s *d* was >0.2 for all single units included in our analysis, which is considered a modest effect size (Cohen, 1988). As there were 6 stimuli presented to each bird, the trial shuffling accuracy yields distributions centered at 16.67% (i.e., “chance” graphed for visual reference; e.g., dashed-line in Fig. 3*F*). In contrast to the *z*-score, which measures how much the stimulus response is relative to baseline across all trials, R_corr_ is a correlation-based metric that takes into account spike-timing variability phenomena such as jitter, missing spikes, and noise in a trial-by-trial basis (Schreiber et al., 2003).

### Code accessibility

The Python code developed for the pattern classifier can be made available on request.

### Statistical analyses

All statistical analyses were performed using IBM SPSS Statistics for Windows (v.23). To test for developmental shifts in multiunit activity, we conducted three-way ANOVAs (phase × hemisphere × stimulus) separately on Trial 1 data (aCSF: *z*-score, firing rates, and classification accuracy). Similar methods were used for testing development changes in single-unit activity. To determine effects of E_2_ on auditory responsiveness, we performed a mixed-effects ANOVA (ME-ANOVA; within-subject factor: treatment; between-subject factors: hemisphere, stimulus). Separate ME-ANOVAs were run for <35-dph versus ≥40-dph subject (see Results). For ME-ANOVAs, we restricted our statistical analyses to aCSF and E_2_ trials (1 and 2, respectively), as we were interested in estrogenic effects on auditory processing; however, we present washout data (Trial 3) in all relevant figures to provide a visual comparison. If a significant interaction was found in the ME-ANOVA model (e.g., significant hemisphere × trial interaction), separate follow-up ME analyses were run for each factor level (e.g., separate analysis for left versus right NCM × trial). All *post hoc* comparisons were performed using Tukey’s honestly significant difference (HSD) test. All statistical tests with *p* < 0.05 were considered significant. See Table 1 for all statistical tests employed for each figure illustrated.

**Table 1.** Statistical table

**Results**	**Data structure**	**Type of test**	**Observed power (α = 0.05)**
Fig. 2*B*, *z*-score	Assumed normal distribution; age (25–34; 40–64; 65–95 dph) × hemisphere (left NCM; right NCM)	Three-way ANOVA	Hemisphere = 0.728; age = 1.00; hemisphere × age = 0.251
Fig. 2*C*, spontaneous firing rate	Assumed normal distribution; phase (sensory; sensorimotor) × hemisphere (left NCM; right NCM)	Three-way ANOVA	Hemisphere = 0.058; phase = 0.738; hemisphere × phase = 0.266
Fig. 2*D*, stimulus-evoked firing rate	Assumed normal distribution; phase (sensory; sensorimotor) × hemisphere (left NCM; right NCM)	Three-way ANOVA	Hemisphere = 0.092; phase = 0.918; hemisphere × phase = 0.626
Fig. 3*C*, *z*-score	Assumed normal distribution; phase (sensory; sensorimotor) × hemisphere (left NCM; right NCM)	Three-way ANOVA	Hemisphere = 0.057; phase = 0.999; hemisphere × phase = 0.105
Fig. 3*D*, classification accuracy	Assumed normal distribution; phase (sensory; sensorimotor) × hemisphere (left NCM; right NCM)	Three-way ANOVA	Hemisphere = 0.051; phase = 0.918; hemisphere × phase = 0.070
Fig. 3*E*, spontaneous firing rate	Assumed normal distribution; phase (sensory; sensorimotor) × hemisphere (left NCM; right NCM)	Three-way ANOVA	Hemisphere = 0.482; phase = 0.815; hemisphere × phase = 0.069
Fig. 3*F*, stimulus-evoked firing rate	Assumed normal distribution; phase (sensory; sensorimotor) × hemisphere (left NCM; right NCM)	Three-way ANOVA	Hemisphere = 0.084; phase = 0.171; hemisphere × phase = 0.078
Fig. 4*A*, *z*-score	Assumed normal distribution; trial (aCSF; E2) × hemisphere (left NCM; right NCM)	Mixed-effects ANOVA	Trial = 0.866; hemisphere = 0.119; trial × hemisphere = 0.182
Fig. 4*A*, inset; *z*-score (rundown)	Assumed normal distribution; trial (trial #1–aCSF; trial #2–aCSF)	Mixed-effects ANOVA	Trial = 0.445
Fig. 4*B*, classification accuracy	Assumed normal distribution; trial (aCSF; E2) × hemisphere (left NCM; right NCM)	Mixed-effects ANOVA	Trial = 0.866; hemisphere = 0.450; trial × hemisphere = 0.369
Fig. 4*C*, spontaneous firing rate	Assumed normal distribution; trial (aCSF; E2) × hemisphere (left NCM; right NCM)	Mixed-effects ANOVA	Trial = 0.997; hemisphere = 0.050; trial × hemisphere = 0.104
Fig. 4*D*, stimulus-evoked firing rate	Assumed normal distribution; trial (aCSF; E2) × hemisphere (left NCM; right NCM)	Mixed-effects ANOVA	Trial = 0.960; hemisphere = 0.185; trial × hemisphere = 0.363
Fig. 5*A*, *z*-score	Assumed normal distribution; trial (aCSF; E2)–separate analyses by hemisphere (left vs. right)	Two-way repeated-measures ANOVA	Left NCM = 0.588; right NCM = 0.303
Fig. 5*B*, classification accuracy	Assumed normal distribution; trial (aCSF; E2)–separate analyses by hemisphere (left vs. right)	Two-way repeated-measures ANOVA	Left NCM = 0.293; right NCM = 0.196
Fig. 5*C*, spontaneous firing rate	Assumed normal distribution; trial (aCSF; E2)–separate analyses by hemisphere (left vs. right)	Two-way repeated-measures ANOVA	Left NCM = 0.629; right NCM = 0.725
Fig. 5*D*, stimulus-evoked firing rate	Assumed normal distribution; trial (aCSF; E2)–separate analyses by hemisphere (left vs. right)	Two-way repeated-measures ANOVA	Left NCM = 0.804; right NCM = 0.758

## Results

### *Distribution of ages* by *hemisphere*

**Figure 2.**
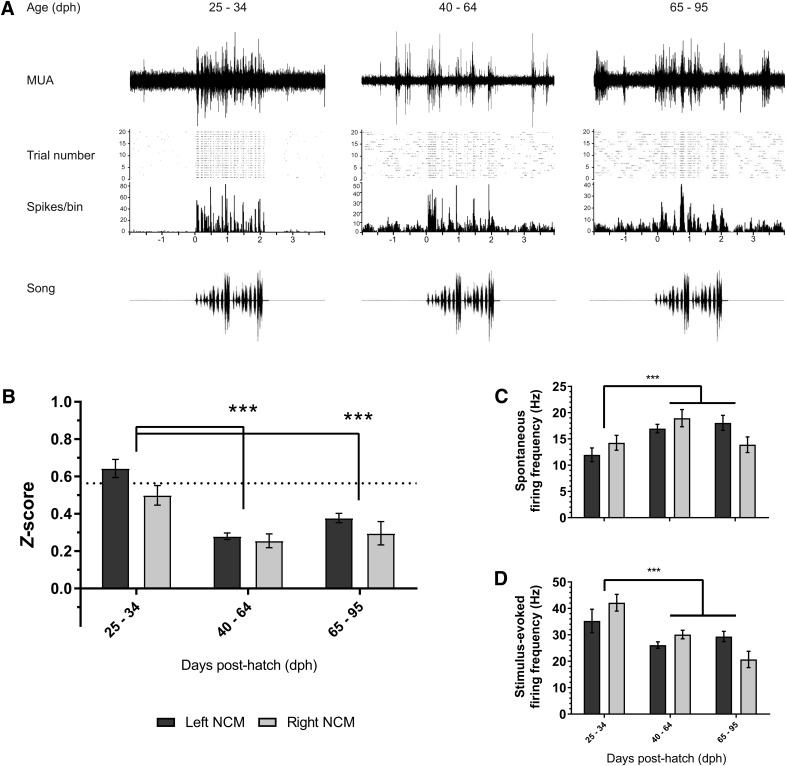
**Multiunit shifts in NCM auditory responsiveness across development. *A***, Representative multiunit recordings from a 25-, 47-, and 95-dph subject (right, left, and left hemisphere, respectively). Top: Representative response to a single presentation of conspecific song (CON2) from a multiunit recording during Trial 1 (aCSF). Middle: Raster plot and corresponding peristimulus time histogram (6-s duration) across all CON2 presentations during Trial 1 (aCSF). Bottom: CON2 sonogram. ***B***, 25–34 dph subjects have higher normalized auditory response than both 40–64 and 65–95 dph birds. Dotted-line in ***B*** is average CON *z*-score from adult male NCM recordings from a separate study (graphed for visual comparison; *n* = 4 birds [195–360 dph; average age = 267.7 dph]). ***C***, ***D***, Based on *z-*score results, we analyzed birds based on critical period phase (sensory [25–34 dph] vs. sensorimotor [40–95 dph]) and found that sensory-aged birds’ NCM have lower spontaneous firing rates (***C***) and elevated stimulus-evoked firing rates (***D***) compared with sensorimotor-aged subjects. ***p < 0.001 (*z*-score: 25–34 dph vs. 40–64 dph, and 25–34 dph vs. 65–95 dph; spontaneous and stimulus-evoked firing: sensory-aged versus sensorimotor-aged). MUA, multiunit activity; CON2, conspecific song 2.

We recorded from 26 unique juvenile male subjects. Of the initial 26 subjects, we obtained 12 successful bilateral recordings. NCM is a bilateral structure with no direct reciprocal connections between hemispheres (Vates et al., 1996), so drug infusions administered to the initial hemisphere are unlikely to directly impact physiology in the contralateral hemisphere. NCM recordings from adult males (≥195 dph) were obtained from a separate set of experiments using identical methods without microdialysis probe (*n* = 4 subjects) to serve as a visual comparison (e.g., Fig. 2*B*).

### Developmental shifts in NCM auditory physiology and encoding

As we were interested in developmental differences in auditory responses, we initially divided our data into three conventional age groups based on their phase in the critical period for song learning (Fig. 1*A*): (1) 25–34 dph (sensory-aged; *n* = 5); (2) 40–64 dph (sensory/sensorimotor-aged; *n* = 13); and (3) 65–95 dph (sensorimotor-aged; *n* = 8); as in Livingston and Mooney (2001).

We first analyzed multiunit recordings to assess whether auditory encoding during baseline conditions (Trial #1; aCSF) differed across subjects depending on the developmental phase and hemisphere (Fig. 2*A*). Multiunit auditory *z*-scores in the left NCM were significantly higher than in the right NCM across development (left: 0.368 ± 0.019; right: 0.340 ± 0.029; mean ± SEM, *F*_(1, 220)_ = 6.663, *p* = 0.010, *η* ^2^ = 0.035). Further, there was a significant age-dependent effect on auditory responsiveness (Fig. 2*B*; *F*_(2, 220)_ = 37.156, *p* < 0.001, *η* ^2^ = 0.275), such that 25–34 dph phase subjects demonstrated significantly higher auditory *z*-scores (0.563 ± 0.037) compared with both 40–64 dph (0.271 ± 0.018; *p* < 0.001) and 65–95 dph subjects (0.349 ± 0.027; *p* < 0.001); there were no significant differences between 40–64 dph and 65–95 dph subjects (*p* = 0.059). There were no significant hemisphere * age interactions for Trial #1 *z*-scores, *F*_(2, 220)_ = 1.464, *p* = 0.233, *η* ^2^ = 0.012. Further, multiunit classification accuracy showed a similar effect of age (*F*_(2, 240)_ = 6.257, *p* = 0.002, *η* ^2^ = 0.059), whereby 25–34 dph subjects had higher accuracies (72.31 ± 2.64%) compared with both 40–64 dph (54.20 ± 2.86%; *p* < 0.001) and 65–95 dph (58.46 ± 4.05%; *p* = 0.001) subjects; 40–64 and 65–95 subjects were statistically similar (*p* = 0.936). No effect of hemisphere on accuracy was observed (*F*_(1, 240)_ = 3.254, *p* = 0.073, *η* ^2^ = 0.016).

As there were no overall age × hemisphere interactions for Trial 1 normalized auditory responses and classification accuracy, and because 40–64 dph and 65–95 dph subjects were statistically similar, we divided subjects into two juvenile age groups for all subsequent analyses: (1) sensory-aged (25–34 dph), and (2) sensorimotor-aged (40–95 dph). This division closely matches a major developmental transition for young male zebra finches, namely before (sensory phase) and after (sensorimotor phase) autogenous singing begins (Clayton, 2013).

Developmental differences in *z*-score can be the result of elevated stimulus-evoked firing rates, reduced spontaneous firing rates, or a combination of both. Therefore, we assessed whether differences in multiunit spontaneous or stimulus-evoked firing frequency in NCM explained elevated *z*-scores in sensory-aged subjects (Fig. 2*C, D*). Sensory-aged subjects had both significantly reduced spontaneous firing (13.246 ± 0.977 Hz) and higher stimulus-evoked firing (39.087 ± 0.2.646 Hz) compared with sensorimotor-aged subjects (spontaneous: 17.432 ± 0.653 Hz, *F*_(2, 222)_ = 11.136, *p* = 0.001, *η* ^2^ = 0.037; stimulus-evoked: 27.295 ± 0.864 Hz, *F*_(2, 222)_ = 11.136, *p* = 0.001, *η* ^2^ = 0.067). The effect of age on spontaneous firing rates was independent of hemisphere (hemisphere: *F*_(1, 222)_ = 1.064, *p* = 0.303, *η* ^2^ = 0.005; hemisphere * age: *F*_(1, 222)_ = 0.509, *p* = 0.477, *η* ^2^ = 0.001). Similarly, no hemisphere * age interactions (*F*_(1, 222)_ = 2.032, *p* = 0.155, *η* ^2^ = 0.005) or overall effect of hemisphere (*F*_(1, 222)_ = 3.092, *p* = 0.080, *η* ^2^ = 0.017) were found for stimulus-evoked firing.

### Developmental shifts in single-unit activity

**Figure 3.**
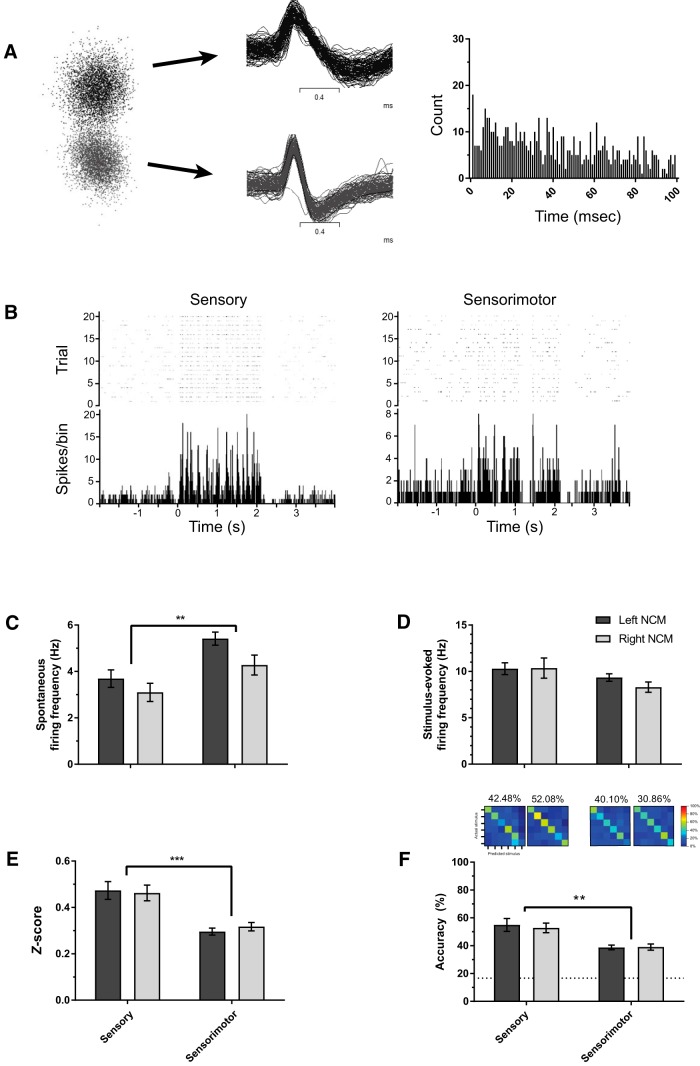
**Single-unit auditory response and encoding in NCM is elevated during sensory phase. *A***, Representative single neurons. Left: Two sorted single units distinctly clustered in principal components space; Middle: 100 sequential iterations from two separate single neurons overlaying their respective wave form template. Right: Interstimulus interval plots for top single unit. Each bin = 1 ms. Units derived from Trial 1 (aCSF) recording from a sensory-aged subject (30 dph; left NCM). ***B***, Raster plot and peristimulus time histogram from representative single units from a sensory-aged and sensorimotor-aged bird (33 [right NCM] and 71 [left NCM] dph). ***C***, ***D***, Spontaneous firing rates are lower in sensory-aged subjects irrespective of hemisphere; however, there are no age-dependent differences in single-unit stimulus-evoked firing rates (***D***). ***E***, ***F***, Across hemispheres, single-unit auditory *z*-scores (***E***) and classification accuracy (***F***) are significantly higher in sensory-aged birds. Dotted-line in ***F*** is chance-level prediction for classifier (1 in 6 chance for accurately classifying a given stimulus = 16.67%). ***p < 0.001; **p < 0.01 (sensory-aged vs. sensorimotor-aged).

While examining multiunit activity provides information about how population of neurons respond to auditory stimuli, we also analyzed isolated single neurons using wave form template matching (Fig. 3*A, B*; see Methods) to investigate whether developmental changes in auditory responsiveness could be explained by the activity of single neurons. Spontaneous firing rates were lower in sensory-aged subjects (3.34 ± 0.28 Hz) compared with sensorimotor-aged subjects (4.91 ± 0.25 Hz; *F*_(1, 292)_ = 8.204, *p* = 0.004, *η* ^2^ = 0.027; Fig. 3*C*). No other significant interactions or main effects were found for spontaneous firing. Stimulus-evoked firing was statistically similar in sensory-aged and sensorimotor-aged juveniles (*p* = 0.315; *η* ^2^ = 0.003; Fig. 3*D*), and there was no effect of hemisphere (*F*_(1, 292)_ = 0.293, *p* = 0.589, *η* ^2^ = 0.001) or hemisphere * age interaction (*F*_(1, 292)_ = 0.239, *p* = 0.626, *η* ^2^ = 0.001). As with the multiunit findings, single units from sensorimotor-aged males had significantly lower *z*-scores (0.310 ± 0.012) compared with units from sensory-aged males (0.461 ± 0.026; *F*_(1, 292)_ = 25.561 *p* < 0.001, *η* ^2^ = 0.080; Fig. 3*E*). There was no effect of hemisphere (*F*_(1, 292)_ = 0.065, *p* = 0.798, *η* ^2^ < 0.001) or hemisphere * age interaction (*F*_(1, 292)_ = 0.469, *p* = 0.494, *η* ^2^ = 0.002) for single-unit *z*-scores.

To evaluate whether developmental changes in communication processing affected auditory encoding, we analyzed the physiology data using a pattern classifier (see Methods). Irrespective of hemisphere, sensory-aged subjects demonstrated higher accuracy rates (53.86 ± 2.50%) compared with sensorimotor-aged subjects (40.38 ± 1.57%; *F*_(1, 262)_ = 11.321, *p* = 0.001, *η* ^2^ = 0.041; Fig. 3*F*). In summary, our findings indicate that auditory neurons in NCM track critical period phase transitions leading to higher auditory responsiveness and coding in sensory-aged, presinging birds.

### Effects of estradiol on NCM physiology and encoding are hemisphere and age dependent

Estradiol enhances stimulus-evoked activity in the NCM of adult male and female songbirds (Remage-Healey et al., 2010, 2012; Remage-Healey and Joshi, 2012). Further, E_2_ production is rapidly enhanced in NCM during social interactions and song playbacks (Remage-Healey et al., 2008). While there are dynamic changes in neuroestrogen synthesis in the NCM of developing songbirds during and after song tutoring (Chao et al., 2015), it is unknown whether E_2_ locally modulates stimulus-evoked activity as in adults. Because we observed clear developmental differences in auditory responsiveness and coding, we elected to analyze subjects separately by age groups for E_2_’s effect on auditory responsiveness.

### Estradiol reduces overall NCM firing in sensory-aged subjects

**Figure 4.**
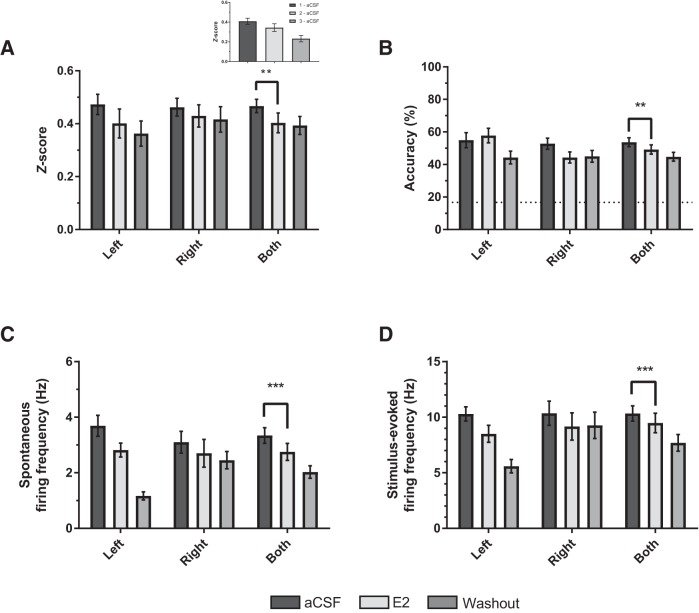
**Estradiol (E_2_) dampens auditory responsiveness in NCM. *A–D***, Relative to aCSF (Trial 1), E_2_ treatment decreased *z*-scores (***A***), classification accuracy (***B***), and spontaneous (***C***) and stimulus-evoked (***D***) firing rates in the NCM of sensory-aged subjects. Hemisphere-specific averages are depicted for visual comparison and consistency, but there was no trial × hemisphere effect. Averaged measurements across hemispheres are depicted in the last set of columns (Both); **p < 0.01 (effect of trial; Trial 1 vs. Trial 2). Dotted-line in ***B*** is chance-level prediction for classifier (1 in 6 chance for accurately classifying a given stimulus = 16.67%). Inset in ***A***, average *z*-score across trials in aCSF rundown experiment (*p* = 0.07; Trial 1 vs. Trial 2; *n =* 5 sensory-aged birds; 6 single units).

Estradiol significantly decreased *z*-scores in sensory subjects (aCSF: 0.461 ± 0.026; E_2_: 0.406 ± 0.035; *F*_(1, 72)_ = 9.659, *p* = 0.003; *η* ^2^ = 0.118; Fig. 4*A*), independent of hemisphere or stimulus (*p* > 0.292). As with normalized auditory responses, E_2_ also reduced spontaneous and stimulus-evoked firing rates (spontaneous: *F*_(1, 72)_ = 23.085, *p* < 0.001; *η* ^2^ = 0.243; stimulus-evoked: *F*_(1, 72)_ = 14.151, *p* < 0.001, *η* ^2^ = 0.164; Fig. 4*C, D*), independent of hemisphere or hemisphere * trial interactions (*p* > 0.05). Further, E_2_ treatment reduced classification accuracy across both hemispheres; *F*_(1, 54)_ = 7.68, *p* = 0.003, *η* ^2^ = 0.153 (aCSF: 51.18% ± 3.35; E_2_: 38.87% ± 1.95; Fig. 4*B*). However, the descriptive data suggest that E_2_’s overall effect on accuracy was influenced by effects in right NCM (Fig. 4*B*; a main effect of hemisphere was nonsignificant, *p* = 0.067). All other main effects and interactions for stimulus and hemisphere were nonsignificant across all physiologic and classification measurements for sensory-aged subjects (*p* > 0.80).

We noted a general trend for attenuated firing rates and *z*-scores across trials for sensory-aged subjects (e.g., compare washout to pre in Fig. 4). Therefore, in a separate set of sensory-aged birds (*n* = 5 birds; 6 single units), we tested whether observed decreases in neural activity also occurred in the absence of E_2_ treatment. To this end, aCSF was administered across all 3 trials in place of E_2_ and a washout trial (Trials 2 and 3, respectively), and resulting activity was compared between Trials 1 and 2. Normalized auditory responses decreased across trials (Fig. 4*A*, inset), but this was not statistically significant (*F*_(1, 30)_ = 3.542, *p* = 0.070; *η* ^2^ = 0.106; Trial 1 aCSF = 0.41 ± 0.03; Trial 2 aCSF = 0.34 ± 0.04), nor were changes in spontaneous firing rates (*F*_(1, 30)_ = 0.473, *p* = 0.497; *η* ^2^ = 0.016; Trial 1 aCSF = 2.55 ± 0.15 Hz; Trial 2 aCSF = 2.37 ± 0.26 Hz). However, there was an overall significant decrease in stimulus-evoked firing (*F*_(1, 30)_ = 5.095, *p* = 0.031; *η* ^2^ = 0.145; Trial 1 aCSF = 7.44 ± 0.56 Hz; Trial 2 aCSF = 5.92 ± 0.78 Hz), and classification accuracy (*F*_(1, 30)_ = 17.075, *p* < 0.001; *η* ^2^ = 0.363; Trial 1 aCSF = 47.92 ± 3.21%; Trial 2 aCSF = 36.55 ± 2.56%) across Trials 1 and 2. There were no significant stimulus * trial interactions or any overall effects of stimulus (*p* > 0.10). Together, results from sensory-aged birds suggest that whereas E_2_ may dampen auditory responsiveness in NCM, this pattern is difficult to disentangle from overall decreases in neuronal firing and classification accuracy in rundown trials with aCSF only.

### Estradiol imparts hemisphere-dependent changes in sensorimotor-aged subjects

**Figure 5.**
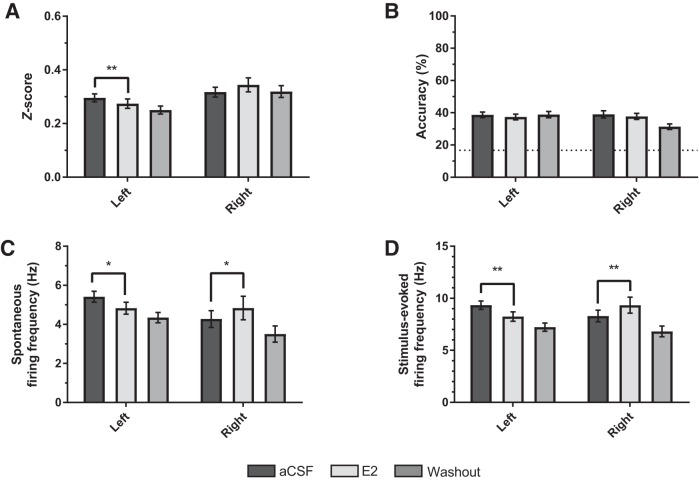
**The effects of estradiol (E_2_) on auditory responsiveness in the NCM of sensorimotor-aged birds are lateralized. *A***, ***B***, Depending on hemisphere, E_2_ treatment either increases (right NCM) or decreases (left NCM) auditory *z*-scores relative to aCSF (Trial 1) in sensorimotor subjects. However, classification accuracy remains unaffected (***B***). ***C***, ***D***, Similar to *z*-scores, both spontaneous (***C***) and stimulus-evoked (***D***) firing rates decrease or increase in response to E_2_ depending on hemisphere (left or right NCM, respectively). Dotted-line in ***B*** is chance-level prediction for classifier (1 in 6 chance for accurately classifying a given stimulus = 16.67%). *p < 0.05 (left/right: Trial 1 vs. Trial 2); **p < 0.01 (left/right: Trial 1 vs. Trial 2).

For sensorimotor-aged subjects, there was a significant trial * hemisphere interaction for *z*-score (*F*_(1, 202)_ = 4.435, *p* = 0.036; *η* ^2^ = 0.021; Fig. 5*A*), such that E_2_ significantly reduced *z*-scores in the left (*F*_(1, 112)_ = 4.845, *p* = 0.030; *η* ^2^ = 0.041) but not in the right (*F*_(1, 90)_ = 2.131, *p* = 0.148; *η* ^2^ = 0.023) hemisphere. Further, E_2_ imparted a hemisphere-dependent effect on firing rates in sensorimotor-aged subjects (spontaneous: *F*_(1, 202)_ = 6.594, *p* = 0.011; *η* ^2^ = 0.032; stimulus-evoked: *F*_(1, 202)_ = 9.426, *p* = 0.002, *η* ^2^ = 0.045; Fig. 5*C, D*). Specifically, E_2_ significantly decreased both spontaneous and stimulus-evoked firing in left NCM (spontaneous: *p* = 0.023; *η* ^2^ = 0.045; stimulus: *F*_(1, 112)_ = 8.066, *p* = 0.005; *η* ^2^ = 0.067), whereas overall firing rates in right NCM were significantly increased (spontaneous: *p =* 0.011; *η* ^2^ = 0.069; stimulus-evoked: *F*_(1, 90)_ = 7.226, *p* = 0.009, *η* ^2^ = 0.074). Classification accuracy was statistically unaffected by E_2_ treatment (*F*_(1, 202)_ = 3.369, *p* = 0.068, *η* ^2^ = 0.016; Fig. 5*B*). In summary, these data suggest that acute modulation of NCM auditory responsiveness by E_2_ is lateralized, and that E_2_ in the right hemisphere of NCM enhances overall neural firing, independent of changes in stimulus coding in sensorimotor-aged birds, whereas the opposite is observed in left NCM.

### Naturalistic sounds elicit higher single-unit auditory responses in NCM across age

In addition to developmental and E_2_ effects on NCM auditory physiology, we compared stimulus-dependent effects on single-unit auditory responsiveness. As work on physiologic preference for natural sounds over synthetic tones in telencephalic auditory forebrain nuclei has been previously reported in several oscine species (Leppelsack and Vogt, 1976; Bonke et al., 1979), including zebra finches (Theunissen et al., 2004; Hauber et al., 2007), we report all the main effects of stimulus in Table *2* for concision. In short, we found that NCM is typically more responsive to naturalistic auditory stimuli (song) compared with a synthetic sound (white noise).

**Table 2.** Stimulus-specific effects on single-unit NCM auditory responsiveness

Dependent variable (single-unit data)	Model	Statistical tests	*F*-values and degrees of freedom	*p*-value	Effect size (partial η^2^)	*Post hoc* results
Development (aCSF; Trial 1 only)						
*z*-score	Phase × hemisphere × stimulus	Three-way ANOVA; Tukey’s HSD	*F*_(7, 292)_ = 4.682	<0.001	0.101	WN < CON1, CON2, HET, JUV CON, and JUV REV CON (*p* < 0.003)
Stimulus-evoked firing	Phase × hemisphere × stimulus	Three-way ANOVA; Tukey’s HSD	*F*_(7, 292)_ = 2.400	0.022	0.054	WN < CON1 and HET (*p* < 0.022)
Classification accuracy	Phase × hemisphere × stimulus	Three-way ANOVA; Tukey’s HSD	*F*_(7, 262)_ = 2.529	0.016	0.063	WN < JUV CON (*p* = 0.023)
Effect of E2 (aCSF vs. E2)						
Sensory						
*z*-score	Trial × hemisphere × stimulus	Three-way ANOVA	*F*_(5, 72)_ = 2.062	0.080	0.125	n/a
Stimulus-evoked firing	Trial × hemisphere × stimulus	Three-way ANOVA	*F*_(5, 72)_ = 1.495	0.202	0.094	n/a
Classification accuracy	Trial × hemisphere × stimulus	Three-way ANOVA	*F*_(1, 54)_ = 1.298	0.278	0.107	n/a
Sensorimotor						
*z*-score						
Left NCM	Trial × stimulus	Two-way ANOVA; Tukey’s HSD	*F*_(7, 112)_ = 3.097	0.005	0.162	WN < BOS, CON1, CON2, and HET (*p* < 0.038)
Right NCM	Trial × stimulus	Two-way ANOVA	*F*_(5, 90)_ = 2.275	0.054	0.112	n/a
Stimulus-evoked firing						
Left NCM	Trial × stimulus	Two-way ANOVA	*F*_(7, 112)_ = 1.365	0.227	0.079	n/a
Right NCM	Trial × stimulus	Two-way ANOVA	*F*_(5, 90)_ = 0.558	0.732	0.030	n/a
Classification accuracy						
Left NCM	Trial × stimulus	Two-way ANOVA; Tukey’s HSD	*F*_(7, 112)_ = 2.415	0.024	0.131	WN < JUV CON (*p* = 0.048)
Right NCM	Trial × stimulus	Two-way ANOVA	*F*_(5, 90)_ = 0.880	0.498	0.047	n/a

n/a, not applicable.

## Discussion

Here, we demonstrate that auditory neurons in pre-singing, sensory-aged male zebra finches have higher auditory responses to natural communication vocalizations compared with older juvenile males. Moreover, sensitivities to E_2_ signaling in auditory cortex change with age: although sensory-aged birds showed an overall decrease in auditory response when treated with E_2_, sensorimotor-aged birds showed a divergent response to E_2_ depending on hemisphere (either overall increase or decrease). Taken together, this study is the first to our knowledge to consider developmental and hemispheric effects on sensory coding and rapid steroid modulation of auditory processing.

### Ontogenetic shifts in vocal communication encoding

During the critical period phase for auditory memory formation, pre-singing (sensory-aged) juvenile songbirds encode communication signals with higher fidelity than juveniles beginning autogenous song production (sensorimotor-aged). As such, elevated auditory-evoked responses in sensory-aged birds suggest the transition from purely auditory encoding (sensory phase) to song production with gradual modification through error-correction (sensorimotor phase) learning may track these perceptual developmental shifts. To our knowledge, this is one of the first studies to document neurophysiological changes in the NCM of pre-singing and sensorimotor learning in juvenile male songbirds. Prior studies have described developmental shifts in the auditory forebrain but have mainly compared 20- versus 35-dph songbirds (all sensory-aged). Amin et al. (2007) described adult-like auditory responses in the brainstem of 20- and 35-dph zebra finches and stimulus-dependent auditory selectivity in the CMM of 35-dph birds. In awake recordings of NCM, electrophysiological auditory responses are comparable at 20 and 30–35 dph (Stripling et al., 2001; Miller-Sims and Bottjer, 2014). Our results build on these findings by expanding the span of time considered during the critical period. These findings inform how learning-dependent transitions during maturation shift auditory processing within NCM.

The elevated auditory processing we observe in sensory-aged subjects may be related to the coincident formation of a tutor auditory memory during this critical period of development. Although auditory input is necessary during the song refinement and error-correction phase in sensorimotor-aged birds (e.g., Mandelblat-Cerf et al., 2014); initially, birds must solely listen before they sing. Perhaps enhanced auditory activity and encoding in NCM during early development ensures a high-fidelity tutor song memory acquisition for young males to subsequently imitate. As NCM is one of the putative loci for tutor song memory (Bolhuis and Gahr, 2006; London and Clayton, 2008; Gobes et al., 2010), elevated auditory responsiveness may be important for early tutor memory consolidation. Alternatively, an increasing amount of tutor experience may facilitate neural transitions from a more broadly tuned auditory circuit (sensory-aged; higher auditory neural activity) to a more selectively tuned circuit (sensorimotor-aged; relatively dampened auditory response). Yanagihara and Yazaki-Sugiyama (2016) found that a relatively short period of tutoring (10 days) radically shifted a subpopulation of single neurons’ auditory selectivity in the NCM of juvenile males and biased neuronal responses primarily toward the tutor and/or birds’ own song. If tutoring experience itself shapes auditory selectivity, then perhaps less experience with tutor or exposure to adult song in general in sensory-aged subjects (9 d relative to onset of critical period opening) compared to older juveniles (15–70 d) explains heightened auditory responsiveness in NCM. However, our finding that stimulus classification accuracy is higher in sensory-aged subjects suggests that rather than NCM being broadly tuned to any sound, young juvenile songbirds can accurately distinguish naturalistic communication signals with higher fidelity than sensorimotor-aged birds.

One caveat to our interpretation that there is a neural “switch point” in auditory processing that precisely tracks behavioral transitions during vocal learning (sensory/pre-singing to sensorimotor/singing) is the ability to dissociate true developmental effects from E_2_-dependent effects. In adult songbirds, song presentation elicits an increase in E_2_ levels in NCM, whereas in juveniles, tutoring leads to decreased E_2_ in NCM and increased levels afterward (Remage-Healey et al., 2008, 2012; Chao et al., 2015). As such, auditory presentations alone may elicit changes in local E_2_ availability that may be age-dependent. However, it remains to be tested whether song presentations to anesthetized songbirds, such as in our study, drive local changes in E_2_ production as with awake, behaving songbirds. Thus, future experiments should clarify whether local E_2_ synthesis in NCM is state-dependent, and should also explore whether local infusion of an aromatase inhibitor during song presentation blocks or unmasks age-dependent and estradiol-dependent regulation of auditory responsiveness in NCM.

Future experiments should also consider these identified developmental milestones in the NCM of juvenile females, who also learn song early posthatching for eventual mate selection in adulthood (Miller, 1979; Riebel, 2000; Terpstra et al., 2006). The extent that elevated auditory responses in NCM of sensory-aged juveniles are similar between males and females will contribute information about its underlying mechanism.

### Acute effects of estrogens on sensory-aged songbirds

Sensory-aged male zebra finches begin forming auditory memories of their tutor’s song before attempting their own vocalizations (Mooney, 2009). As such, we predicted that E_2_ would enhance auditory tuning as it does in adults (Remage-Healey et al., 2010, 2012; Pinaud and Tremere, 2012; Remage-Healey and Joshi, 2012; but see Lattin et al., 2017). However, E_2_ treatments led to significant decrements in auditory processing irrespective of hemisphere. One explanation may be that E_2_ dynamics change during development. Chao et al. (2015) observed acute decreases in E_2_ levels during tutoring in the NCM of developing male zebra finches, but also that NCM E_2_ levels increase immediately after a tutoring session. As such, acute neuroestrogen production may impair auditory memory acquisition during a learning session in sensory-aged songbirds (Korol and Pisani, 2015; Rensel et al., 2015), whereas post-training E_2_ increases may facilitate memory consolidation (Srivastava et al., 2013; Frick, 2015; Vahaba and Remage-Healey, 2015). Further, the expression of telencephalic GPER1 (G-protein coupled estrogen receptor 1 that can mediate rapid neuroestrogen signaling [Rudolph et al., 2016]) is five-fold higher in sensory-aged zebra finches (Acharya and Veney, 2011). Therefore, NCM may be particularly sensitive to low concentrations of E_2_ in sensory-aged animals. This work thus suggests that dynamic changes in estrogen receptor and aromatase protein expression in NCM across development may explain an initial suppressive effect of E_2_ signaling on auditory processing in sensory-aged male songbirds.

One important caveat to these results is that in a separate set of sensory-aged birds with aCSF retrodialyzed across all three trials (run-down experiment), we observed decreased classification accuracy and stimulus-evoked firing rates, as well as a trend for reduced normalized auditory responsiveness. These results make it more difficult to disentangle the effects of E_2_ on decreases in NCM responsiveness and encoding in sensory-aged subjects from purely time-dependent effects. Nonetheless, E_2_ reduced spontaneous firing in sensory-aged birds, which was not observed in aCSF-only trials, and may reflect a true dampening of auditory responsiveness. Moreover, the run-down experiment emphasizes how our observations of increased firing during E_2_ treatment, as seen in the right NCM of sensorimotor-aged subjects, are likely counteracting this overall steady run-down effect in juvenile males.

### Acute, lateralized effects of estrogens on sensorimotor-aged songbirds

The lateralization of E_2_ actions on auditory encoding and firing rate in NCM differ across development. In sensorimotor-aged birds, E_2_ imparts a hemisphere-dependent effect. In left NCM, E_2_ led to decreased normalized auditory response, as well as spontaneous and stimulus-evoked firing rates, without affecting classification accuracy. In contrast, E_2_ administration in the right NCM increased stimulus and spontaneous-evoked firing rates, without impacting normalized auditory responses or classification accuracy. These data add to a growing literature on the lateralized neuromodulation of hearing by brain hormones. For example, oxytocin receptors are preferentially upregulated the in left auditory cortex of maternal female rats, which enhances pup call saliency/encoding (Marlin et al., 2015). In male European starlings, inhibiting aromatase suppresses vocal communication responses in the left, but not right, hemisphere of the auditory forebrain (De Groof et al., 2017). Similarly, blocking E_2_ synthesis in left but not right NCM extinguishes male songbirds’ behavioral preference for their own song (Remage-Healey et al., 2010). Therefore, our findings add further evidence for hemisphere-dependent hormone neuromodulation of communication processing in auditory cortex, and expand this concept to include developing animals.

Prior work on developmental neuromodulation has not addressed how sensitivities to E_2_ may differ by hemisphere, and whether estrogen synthase or estrogen receptor expression is similarly lateralized. Chao et al. (2015) found decreased E_2_ in NCM during tutor song exposure in developing male subjects; however, E_2_ was measured only within the left NCM. Therefore, our current results suggest that E_2_ fluctuations in right NCM may increase or remain unchanged during tutoring. Future experiments should also clarify changes in aromatase and estrogen receptors (both nuclear [ERα and ERβ] and membrane-bound [GPER1; mGluR1/ERα]) across development and between hemispheres, as these factors may also account for divergent effects of E_2_ on auditory physiology in NCM across the critical period. Alternatively, the auditory cortex of juvenile male zebra finches may mature at different rates depending on hemisphere. Our data suggest that the right NCM matures faster than the left, as E_2_ enhancement of auditory responsiveness is more adult-like in the right versus left NCM of sensorimotor-aged subjects (Remage-Healey et al., 2010). Future experiments exploring developmental changes should also identify whether NCM is lateralized in neuronal development across the critical period, as well, since there are no reported differences in NCM cell density between developing versus adult male NCM (Stripling et al., 2001), nor any published quantifications of left versus right neuronal density in NCM at any age.

These findings contribute to a broader point of interest on how steroid hormones may participate in learning. Accumulating evidence demonstrate that rapid, local E_2_ synthesis and signaling is critically linked to neural plasticity in the hippocampus and amygdala (Zhao et al., 2010; Srivastava et al., 2013; Bailey et al., 2017; Bender et al., 2017). Less is known about rapid E_2_ signaling and plasticity in sensory cortices, such as the auditory cortex. In adult zebra finches, blocking global E_2_ synthesis impairs neural adaptation to familiar songs in NCM, a proxy for auditory memory formation (Yoder et al., 2012). In juvenile songbirds, circulating E_2_ predicts tutor imitation accuracy (Marler et al., 1987); however, the majority of studies on hormones and song learning in development have focused on androgens. Administering testosterone or dihydrotestosterone to juvenile songbirds prematurely crystallizes song (Korsia and Bottjer, 1991; Bottjer and Hewer, 1992; Whaling et al., 1995; Livingston and Mooney, 2001; however, see Templeton et al., 2012). Therefore, it remains to be tested how neuroestrogen synthesis in the auditory forebrain is involved in vocal learning. Our results suggest that local E_2_ may interfere with auditory encoding in sensory-aged birds and within the left NCM of sensorimotor-aged birds, whereas E_2_ in the right NCM in sensorimotor-aged animals may aid in encoding song. These possibilities await future experimental tests to determine potential functional roles for E_2_ in song learning.

## Conclusion

Here, we demonstrate that robust shifts in sensory processing in the auditory cortex precisely track experience-dependent critical period milestones, and extend our understanding of estrogen-dependent neuromodulation of auditory responsiveness across development. Our findings indicate that age and hemisphere are critical factors to consider when evaluating sensory physiology in development and in response to neuromodulators. Further, these data provide insight into a broader understanding of how estrogen signaling and audition may change across the lifespan, and in relation to hemisphere and communication learning. In humans, estrogens generally enhance hearing in adulthood, and appear to have a lateralized effect on listening (Tillman, 2010) and verbal memory (Fernandez et al., 2003). Interestingly, Wild et al. (2017) found adult-like neural responses to speech in the auditory cortex of 3- and 9-month-old infants, a time during which circulating estrogen levels predict future language success (Wermke et al., 2014; Quast et al., 2016). As such, future research should consider both hormonal state and hemisphere when studying hearing-evoked neural changes in auditory cortex.

## Synthesis

Reviewing Editor: Jeffrey Blaustein, University of Massachusetts Decisions are customarily a result of the Reviewing Editor and the peer reviewers coming together and discussing their recommendations until a consensus is reached. When revisions are invited, a fact-based synthesis statement explaining their decision and outlining what is needed to prepare a revision will be listed below. The following reviewer(s) agreed to reveal their identity: Colin Saldanha, Eric Fortune.

There was a good deal of enthusiasm for your manuscript, and the changes needed are all relatively minor.

Reviewer 1 made the suggestion that a caveat be included in the discussion explaining the possibility that the developmental changes may reflect hormonal effects. In addition, standard nomenclature for estradiol is with 2 as subscript.

Reviewer 2 suggests that an explanation be given for the separate analyses. This reviewer also points out the problem with the phrase “approached significance.” While this reviewer suggests use of the term “trend,” this too should be avoided. There has been quite a bit written and discussed recently about the arbitrariness of p <.05. However, if it is used, it should be adhered to. Perhaps in the discussion, in your overall model, you could mention these “trends.”

Reviewer 2 also suggests refocusing the manuscript. This seems like a good suggestion, but we will leave this decision up to you. This reviewer also suggests eliminating the statements about human health. I see no harm in retaining these, since the work is funded by NIH. The reviewer made a number of suggestions to improve the writing; we agree with these.

I am including the full reviews for your information.

Reviewer 1:

The present submission describes a set of experiments aimed at understanding the neural and hormonal correlates of song learning in male juvenile zebra finches. The researchers target a key transition in song-learning behavior (sensory to sensorimotor), and use electrophysiology and microdialysis to measure changes in physiological properties of neurons in the caudomedial nidopallium (NCM) and the effects of E2 provision on these characteristics. They report several important changes in the firing patterns of NCM neurons as birds' transition into the sensorimotor phase including but not limited to: left hemisphere biases in auditory properties of NCM neurons, and a decrease in auditory responsiveness with increasing age. This decrease apparently stems from reduced spontaneous firing rates and higher firing in response to stimuli. This general pattern was upheld in putative single-neuron firing rates as evidenced by decreases in spontaneous firing.

Since neuroestrogens have been implicated in auditory responsiveness to song and to singing behavior itself, the authors then tested the role of E2, microdialyzed directly into the NCM and found a corresponding E2-dependent decrease in auditory responsiveness in sensory birds. Local estrogens appear to have a complex effect on older birds, with interesting laterality and amplification effects.

This is a strong paper that addresses a very important issue. The authors' are to be congratulated for the technical proficiency inherent in successfully collecting these data. Chronic recording in birds with a less than solid skull cannot be easy. The data are well presented and the overall story hangs together quite well.

While the endocrine effects on NCM neurons appears to be solid, and “matches,” in a general sense the developmental changes described, I wonder if the latter are true developmental changes, or a reflection of hormonal influences on NCM neurons. The very first paper describing microdialysis of E2 in the NCM shows quite clearly that the area around the probe (and I presume the area around a chronic recording probe) is covered with glial cells that seem to express the enzyme aromatase (see Remage-Healey et al. Nature Neurosci, 2008, Figure 1A). In other words, I believe that all birds with chronic probes would have plenty of E2 available to the NCM as a function of glial provision of estrogens perhaps via the aromatization of circulating androgens. That being said, the developmental changes described may reflect E2-dependent effects and not necessarily the intrinsic developmental changes in the properties of neurons.

Separating true developmental and E2-dependent effects may be extremely difficult, but could be achieved by recording NCM neurons in the presence of an aromatase inhibitor. This would remove aromatase-dependent effects on electrophysiological properties of NCM neurons and perhaps reveal the underlying less adulterated developmental changes. Since the authors do an excellent job in describing the role of E2 in song learning (Discussion), I believe conducting the experiment above is beyond the scope of this manuscript (although it may be a good experiment in the future?). However, adding a strong caveat to the Discussion describing the possibility that the developmental changes described may reflect hormonal effects, and a manner of addressing this potential confusion - is in order.

Minor point. I believe estradiol is denoted as E2 (subscript) and not E2.

Reviewer 2:

This manuscript describes changes in spiking responses of neurons in NCM to playback of sound stimuli in relation to developmental stage and local application of estrogens. In addition, through the use of bilateral recordings, the authors investigated potential lateralization of these changes. Younger birds exhibited stronger auditory responses than older birds. In older birds, the effects of E2 application were lateralized - E2 reduced responses in left NCM and enhanced responses in right NCM. In sum, local application of estrogen has both age and hemisphere-dependent effects on auditory processing.

Major comments

1) I'm not sure what the point of the separate analysis of multi-unit and single-unit data. Certainly reviewers in the birdsong community are known to be both demanding and rather inconsistent in their demands for these approaches to analyzing physiological data. I suspect that the approach used in the manuscript was done specifically to avoid potential problems with reviewers. But there needs to be some explanation of why these two analyses of the same data were performed, and a direct statement of the differences/similarities between the analyses. Please make edits to ensure that this is obvious to your readers.

2) There are several instances where the phrase “approached significance” is used. This is not appropriate and should be removed. Recall that the cutoff of 0.05 is a rather arbitrary point on a curve, and that even with 0.05, you expect that some correlations will occur at random rather than be a result of a causal relation. A better and more common way to describe these sorts of results that happen to be near the arbitrary limit for significance is to call them a trend. This wording is also wrong, but is commonly used and perhaps less wrong than saying that a result approaches significance. Recall that the goal is to identify the nature of functional relationships in the brain - which is complicated by the fact that firing activity is variable by nature.

In sum, the use of the phrase “approached significance” is quite unusual in my experience, and highlights the central problem that is routinely leveled against the use of p values. Further, I'm not sure what point you are trying to make by identifying these trends. Perhaps a better approach would be to explain the results in more detail, beyond the focus of statistical tests. Another, even better approach would be to generate a model that captures your results, including the variation.

3) The manuscript is not well-focussed on the main results. The central result, that there are hemispheric differences in the effects of E2 application in the sensorimotor-aged birds is interesting. The introduction is focussed on critical periods, which is orthogonal to this main result. This manuscript can be improved by a rewrite with the aim of focussing the text on the main results rather than the more general approach.

Minor comments

ABSTRACT

1) Please change “nidopallium (NCM) is a higher-order brain region” to “nidopallium (NCM) is a telencephalic brain region”. Higher-order is sometimes used in publications, but it has no real meaning.

2) Please change “Thus, we sought to” to “We sought to” The experiment is not a direct consequence of the previous results, so drop the word “thus”.

3) I'm not sure how to parse the sentence that starts with “Further, we observed...” and ends with “was observed in controls.” Is the goal here to suggest that the controls and the treated birds just sit on either side of the 0.05 cutoff? That seems to weaken your claim. And I'm not sure about the context of this result.

4) Please change “E2 either dampened or enhanced auditory responsiveness in left or right NCM, respectively.” to “E2 dampened responsiveness in left NCM but enhanced responsiveness to auditory stimuli in right NCM.”

5) The significance statement is unusually weak. It seems to suggest that the most important message of the paper is highlighting the importance of considering hormones and hemispheres when evaluating auditory responses. Highlight your result more directly. And, the last two sentences that tie the work to human health are probably not necessary.

INTRODUCTION

1) The first two sentences of the Introduction focus on Critical Periods, which is not mentioned in the Abstract. I also see the issue of visual critical periods to be a distraction from the main result. I doubt that you think that the importance of your work is that you are applying lessons learned from the visual system to explore the auditory system. Rather, your paper is about the effects of hormones during different stages of behavioral development. Please rewrite to better focus on your work and the body of related works.

2) Please change “Circulating estrogens fluctuate markedly across the critical period” to “Circulating estrogens fluctuate across the critical period” or rework this sentence further.

3) Please change “Therefore, we tested two hypotheses” to “We tested two hypotheses”

MATERIALS AND METHODS

1) Please change “Brains were carefully dissected out and placed” to “Brains were removed and placed” The word carefully is not necessary because I can hardly imagine that you removed the brains in a careless manner. And “dissected out” is informal jargon.

RESULTS

1) This requires more explanation: “there are no reported direct reciprocal connections between NCM”.

2) Please change “methods sans microdialysis” to “methods without microdialysis”

3) Throughout the manuscript, please use one term each to refer to sensory-aged and sensorimotor-aged subjects. Sometimes the animals are called sensory subjects and sometimes sensory-aged subjects. Pick one and stick with it everywhere for clarity. I prefer “sensory-aged subjects” over “sensory subjects” to disambiguate the use of the word “sensory” in the manuscript.

4) If selectivity is observed in multiple unit recordings, then the single units can only be the same or more selective. In this way, the statement “To investigate whether age-dependent differences in firing rates observed at the population-level were maintained at the single-unit level” is wrong. Please edit.
